# Genetic structure and diversity of *Mycoplasma hyopneumoniae* based on a MLVA typing scheme

**DOI:** 10.3389/fvets.2024.1510825

**Published:** 2025-01-15

**Authors:** Pablo Tamiozzo, Virginia García, Raúl E. González-Ittig, Maria Pieters

**Affiliations:** ^1^Departamento de Patología Animal, Facultad de Agronomía y Veterinaria, Universidad Nacional de Río Cuarto, Córdoba, Argentina; ^2^Cátedra de Genética de Poblaciones y Evolución, Facultad de Ciencias Exactas, Físicas y Naturales, Universidad Nacional de Córdoba, Córdoba, Argentina; ^3^Instituto de Diversidad y Ecología Animal (IDEA; CONICET-UNC), Córdoba, Argentina; ^4^Department of Veterinary Population Medicine, College of Veterinary Medicine, University of Minnesota, Saint Paul, MN, United States; ^5^Veterinary Diagnostic Laboratory, College of Veterinary Medicine, University of Minnesota, Saint Paul, MN, United States; ^6^Swine Disease Eradication Center, College of Veterinary Medicine, University of Minnesota, Saint Paul, MN, United States

**Keywords:** *Mycoplasma hyopneumoniae*, genetic structure, panmictic-clonality, MLVA, porcine enzootic pneumonia, admixed ancestry

## Abstract

**Background:**

Several epidemiological studies have been carried out using Multiple-Locus Variable-number tandem repeat Analysis (MLVA) for *M. hyopneumoniae* typing. However, a global perspective on the implications of the genetic diversity of this pathogen is lacking.

**Objective:**

This study aimed to determine and to analyze the genetic structure of *M. hyopneumoniae* based on the *p97*R1-*p146*R3 MLVA typing scheme and to characterize, analyze and compare MLVA types among countries where the information was publicly available.

**Methods:**

A set of 797 publicly available data of *M. hyopneumoniae* *p97*R1-*p146*R3 MLVA genetic types from six different countries were analyzed using Genalex 6.41 software to characterize loci polymorphism and using Structure 2.3.4 software in order to identify the genetic structure.

**Results:**

A total of 185 MLVA types were identified among the analyzed data. For the *p97*R1 and *p146*R3 loci, most of the molecular variation in *M. hyopneumoniae* populations was identified within countries. Three genetic clusters and their recombinations were identified globally.

**Conclusion:**

*M. hyopneumoniae* is a genetically diverse pathogen with limited clonality and three well-defined clusters and their combinations were identified in this investigation. The greatest genetic variation of *M. hyopneumoniae* was observed within countries.

## 1 Introduction

*Mycoplasma hyopneumoniae* is the primary agent involved in porcine enzootic pneumonia and the Porcine Respiratory Disease Complex. Infections with this bacterium are highly prevalent in almost all swine-producing areas, causing significant economic losses to the pig industry worldwide (1). Information on the genetic diversity of *M. hyopneumoniae* can have implications in the design, identification, and application of pig health intervention strategies, such as emergence of new and drug-resistant strains, development of precision diagnostics, therapeutics and vaccines, as well as epidemiological studies.

To identify genetic variability in *M. hyopneumoniae*, different typing schemes have been reported, such as Random Amplification of Polymorphic DNA [RAPD-PCR (2)], Amplified Fragment Length Polymorphism [AFLP (2, 3)], Arbitrarily Primed-PCR [AP-PCR (4)] and Pulsed Field Gel Electrophoresis [PFGE (5)]. Although some of the above mentioned techniques have demonstrated high discriminatory power, currently, they are not widely used. In addition, Multiple-Locus Sequence Typing (MLST) and Multiple-Locus Variable-Number Tandem Repeat Analysis (MLVA) have been or are used for genetic typing of this microorganism worldwide (6–9, 43, 45, 46). While MLST can be laborious, costly, and has mainly been employed for research purposes (10–14), MLVA can be applied with ease, is less expensive, and has been increasingly used for *M. hyopneumoniae* typing (7, 9, 15, 16), even in field based investigations (17, 43). From a proposed repertoire of twenty-two Variable Number Tandem Repeats (VNTR) regions, four have proven to be highly polymorphic (6), namely *p97*R1, *p146*R3, H4 and H5. Although analyzing the four loci of the MLVA scheme has shown a high discriminatory power (6, 18), the detection of different *M. hyopneumoniae* genetic types has been evidenced using three (15, 19), two (7) or even one VNTR (20, 21).

Numerous epidemiological studies have been carried out using different MLVA schemes in various countries, at farm, regional and/or country level (7, 15–17). However, a global perspective on the implications of the genetic diversity of this etiological agent is lacking. To this extent, *M. hyopneumoniae* has been pointed out as a microorganism with limited clonality (13). Notwithstanding, information is needed on the overall allele frequency, distribution of MLVA types, and genetic structure of this pathogen. Therefore, the objectives of this study were to determine and to analyze the genetic structure of *M. hyopneumoniae* based on the *p97*R1-*p146*R3 MLVA typing scheme and to characterize, analyze and compare MLVA types among countries where the information was publicly available.

## 2 Materials and methods

### 2.1 Study inclusion criteria and data extraction

Considering that the majority of published studies using MLVA as a typing scheme for *M. hyopneumoniae* included the *p97*R1 and *p146*R3 loci, and that both genes encode well-known adhesins (22–24), only studies in which at least these two loci were typed were included in this investigation. Moreover, this criterion was applied as it has been previously suggested that two loci are to be the minimum in *M. hyopneumoniae* typing schemes (25). Additionally, a small number of reports using three or four loci were available in the literature. The data set used in this investigation also included unpublished data from our research group. From the selected studies, only tandem repeats from the *p97*R1 and *p146*R3 loci were included. Table 1 shows the number of VNTR types according to country. Thus, 797 VNTR type data sets (VNTR types, from hereon) were recovered and analyzed.

**Table 1:** Variable number tandem repeat data collected by country, according to the reference.

Country	Number of VNTR data collected	Reference
Argentina	10	(19)
	27	(15)
	28	This study
Belgium	31	(9)
Brazil	95	(7)
	103	(47)
Mexico	26	(7)
Spain	25	(7)
	44	(18)
United States	209	(7)
	95	(48)
	41	(17)
	63	This study

### 2.2 Data analysis

The number of alleles (Na), number of effective alleles (Ne), Shannon’s Information Index (I) and values of diversity (h) and unbiased diversity (uh) were calculated using the program Genalex 6.41 (26) as a way to characterize the polymorphism of *M. hyopneumoniae* loci in each country for which data was available. The pairwise differentiation among countries using the Nei unbiased genetic distance was also calculated using the above-mentioned program and the allele frequencies by population and by locus was plotted. Analysis of molecular variance (AMOVA) among countries, regions (America vs. Europe), and within populations (countries), was performed for both loci, separately and together.

The genetic structure of *M. hyopneumoniae* was examined using the Bayesian algorithm implemented in Structure 2.3.4 (27) assuming a model of population admixture and correlated allele frequencies among groups. The analysis was performed without the inclusion of any geographic information. Thus, the software could identify the most likely number of genetic clusters without *a priori* information. Analyses for a number of K ranging from 1 to 7, for 2,000,000 iterations, including a burn-in of 500,000 iterations was performed. The parameter Alphapropsd = 0.125 to improve mixing was used. The procedure was repeated five times for each K to check for consistency. The optimal value of K was calculated according to the ΔK method described by Evanno et al. (28) using the online Structure Harvester 0.6.7 software.^1^ Replicate runs of Structure were analyzed using Clumpp 1.1.2 (29) to summarize each type’s membership across runs using a greedy algorithm. Results were averaged to build summary bar plots for each VNTR type using the program Distruct 1.1 (30). For a VNTR type to be considered part of a cluster, the membership to one cluster should had been more than 0.90, otherwise, admixed ancestry was assumed (31).

## 3 Results

**Figure 1:**
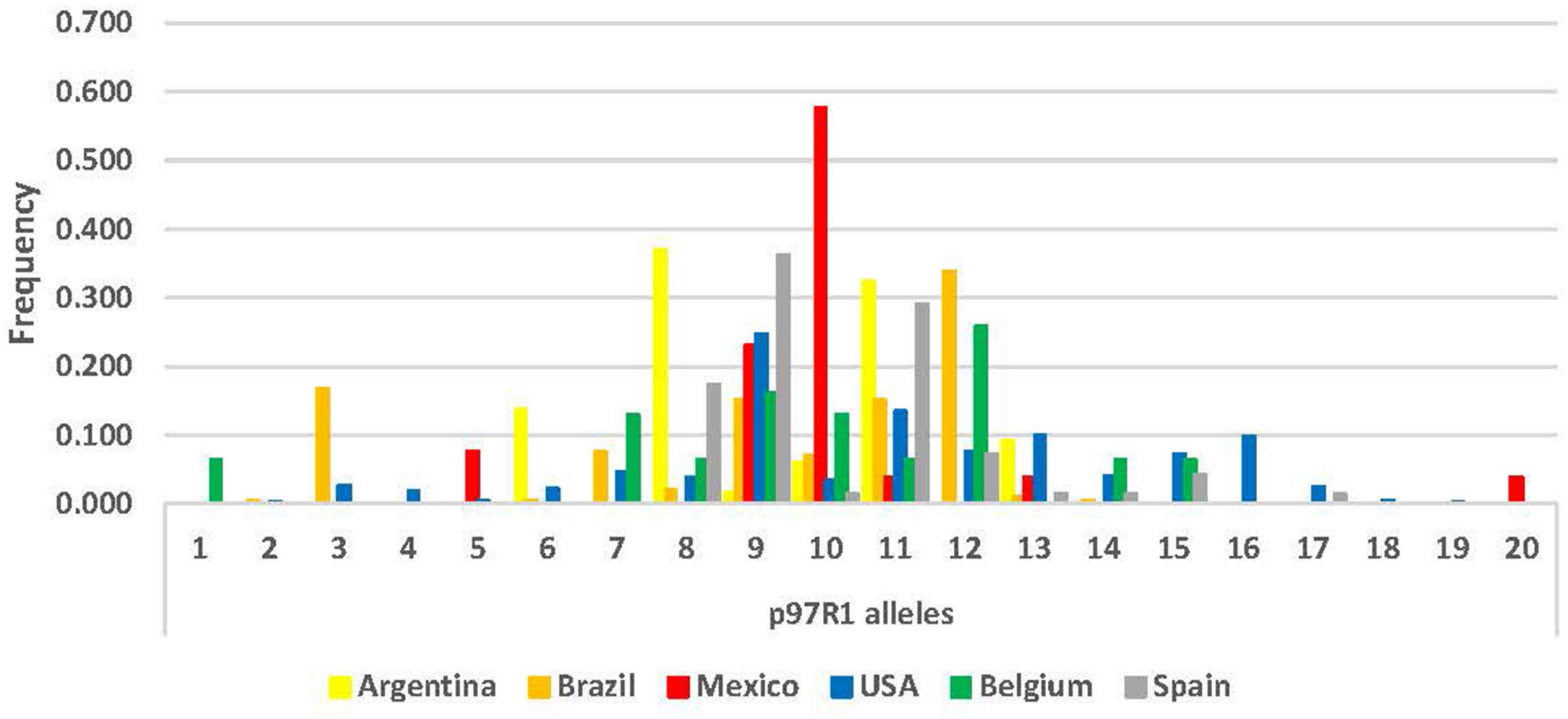
Allele frequency distribution for *p97*R1 locus, according to number of Tandem Repeats (TR), by country.

**Figure 2:**
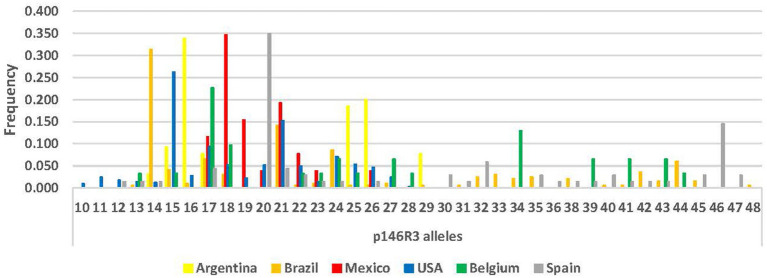
Allele frequency distribution for *p146*R3 locus, according to number of Tandem Repeats (TR), by country.

Locus *p97*R1 revealed 20 alleles, while locus *p146*R3 revealed 38 alleles, with tandem repeats (VNTRs) ranging from 10 to 48 (Supplementary Table S1; Figures 1, 2). For *p97*R1, the VNTR types with 9, 10 and 11 tandem repeats were identified in all countries, while unique alleles were reported in the United States (VNTRs 4, 16, 18 and 19), Belgium (VNTR 1), and Mexico (VNTR 20). For *p146*R3, the only type shared in all the countries was VNTR 17, while unique types were observed in the United States (VNTRs 10 and 11), Spain (VNTRs 30, 36, 46 and 47), and Brazil (VNTRs 33 and 48) (Supplementary Table S1). For *p97*R1, the country with the highest number of alleles (Na), effective alleles (Ne) and most diversity (h) and unbiased diversity (uh) by population was the United States (Table 2). For *p146*R3, the country with the highest number of alleles (Na) was Brazil. The highest number of effective alleles (Ne) and the most diversity (h) and unbiased (uh) diversity by population was Belgium (Table 2).

**Table 2:** Sample size (N), Number of alleles (Na), Number of effective alleles (Ne), information index (I), Diversity (h) and Unbiased Diversity (uh) by country (population) for the *p97*R1 and *p146*R3 loci.

Country	Locus	N	Na	Ne	I	h	uh
Argentina	*p97*R1	65	6	3,671	1,462	0,728	0,739
	*p146*R3	65	7	4,763	1,722	0,790	0,802
Belgium	*p97*R1	31	9	6,816	2,056	0,853	0,882
	*p146*R3	31	15	9,515	2,486	0,895	0,925
Brazil	*p97*R1	198	11	5,012	1,825	0,800	0,805
	*p146*R3	198	27	7,082	2,509	0,859	0,863
Mexico	*p97*R1	26	6	2,522	1,229	0,604	0,628
	*p146*R3	26	8	4,899	1,795	0,796	0,828
Spain	*p97*R1	69	9	3,944	1,603	0,746	0,757
	*p146*R3	69	24	6,373	2,499	0,843	0,855
United States	*p97*R1	408	18	8,388	2,411	0,881	0,883
	*p146*R3	408	19	8,212	2,471	0,878	0,880

A total of 185 *M. hyopneumoniae* typologies were identified among the 797 VNTR types analyzed. The most frequent VNTR types (*p97*R1-*p146*R3, respectively) were: 9–15 (8.4%), 11–21 (7.4%), 14–14 (6.5%), 13–17 (3.4%), 9–20 (3.3%), 16–24 (2.9%) and 8–16 (2.4%), which were present in all countries included in the study, except Belgium (Supplementary material S2). The remaining VNTR types were identified in less than 2.1% of the analyzed data. The 44.3% (82/185) of the identified types were deemed unique and were distributed in all countries (Supplementary material S2). In Argentina there were 13 VNTR types, being the most frequent 8–16 (27.7%) and 11–26 (15.4%). In Belgium, 20 types were recognized, with 12–17 (19.4%) and 7–34 (9.7%) being the most prevalent. In Brazil 52 typologies were identified, the most frequent: 12–14 (24.2%) and 11–21 (13.6%). In Mexico 12 types were observed, with 10–18, 10–19 and 10–17 in 30.8, 15.4 and 11.5%, respectively. In Spain 32 types were identified, including 9–20 (34.8%) and 11–46 (14.5%). In the United States a total of 101 types were observed, the most prominent being 9–15 (16.2%) and 11–21 (7.6%) (Supplementary material S2). All MLVA types identified in this investigation were not present in all countries, with no type present in more than three countries.

Similarity coefficients of Nei’s unbiased genetic distance between *M. hyopneumoniae* populations ranged from 0.471 to 1.943 (Table 3). The highest distance was observed between Mexico and Argentina (1.943%), while the highest Nei unbiased genetic identity was observed between Brazil and Belgium (0.6%; Table 4).

**Table 3:** Pairwise population matrix of nei unbiased genetic distance.

	Argentina	Brazil	Mexico	United States	Belgium	Spain
Argentina	0,000					
Brazil	1,480	0,000				
Mexico	1,943	1,199	0,000			
USA	0,882	0,643	0,836	0,000		
Belgium	1,298	0,377	0,604	0,471	0,000	
Spain	0,894	0,890	1,233	0,521	0,759	0,000

Shading indicates de maximum distance.

**Table 4:** Pairwise population matrix of nei unbiased genetic identity.

	Argentina	Brazil	Mexico	United States	Belgium	Spain
Argentina	1,000					
Brazil	0,228	1,000				
Mexico	0,143	0,301	1,000			
USA	0,414	0,526	0,434	1,000		
Belgium	0,273	0,686	0,546	0,624	1,000	
Spain	0,409	0,411	0,291	0,594	0,468	1,000

Shading indicates the maximum similarity.

Results of the AMOVA indicated that, for both loci, most of the molecular variation in *M. hyopneumoniae* populations (65%), existed within populations (countries), with less variation between regions (28%) and among populations (7%). For the *p97*R1 locus, most of the variation existed within populations (90%) and 10% among populations, with no difference between regions. For the *p146*R3 locus, most of the variation existed within populations (61%), with less variation among regions (32%) and populations (7).

**Figure 3:**
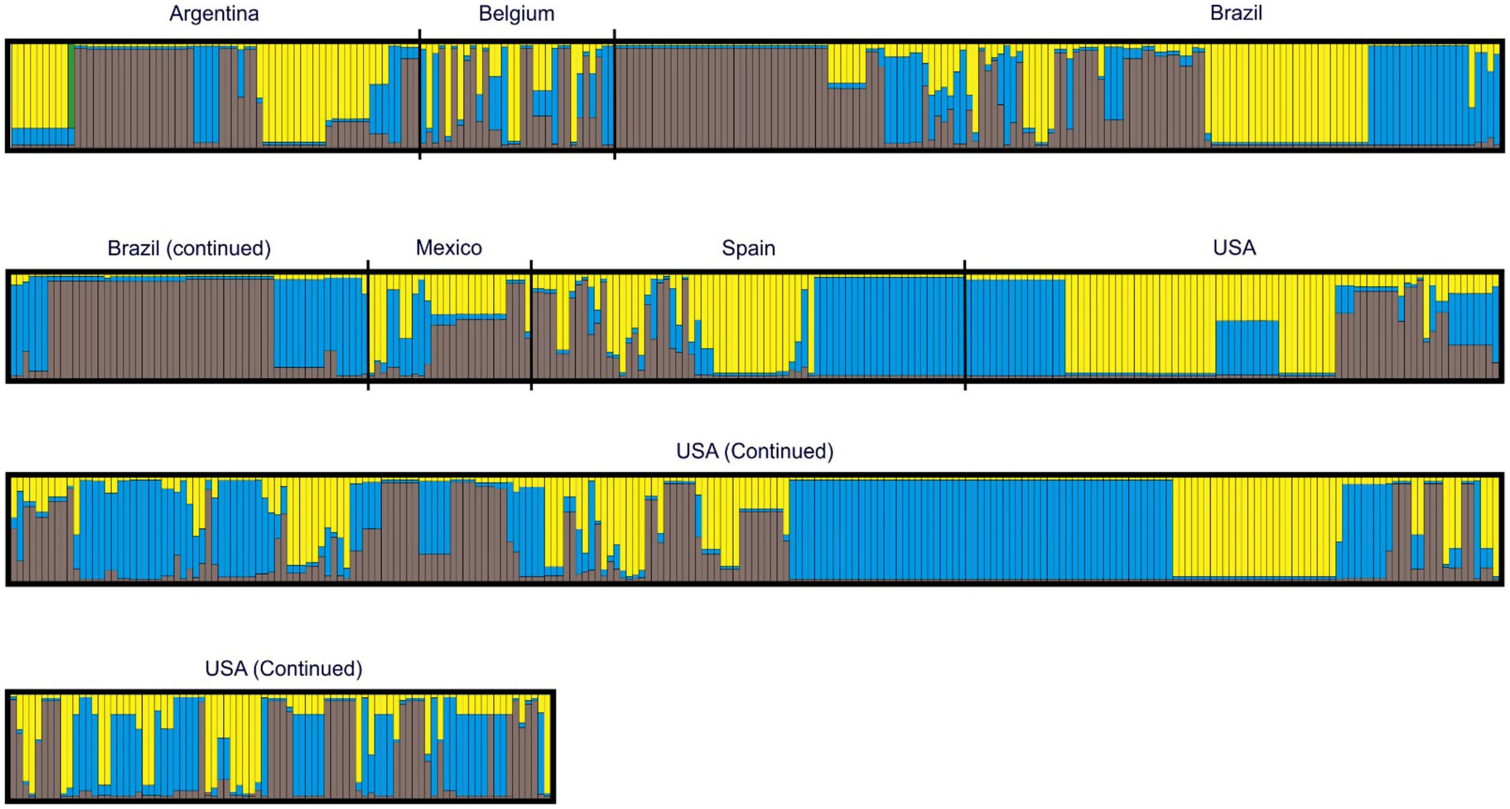
*Mycoplasma hyopneumoniae* individual Bayesian assignment probabilities of *p97*R1-*p146*R3 MLVA using the program STRUCTURE for k clusters. Each vertical line represents the probability of an individual belonging to one of three clusters (represented by different colors) or a combination if ancestry is mixed.

Analyses revealed three genetic clusters, with log posterior probabilities of −3611.72 ± 2.83 for the 797 types (Figure 3). Different countries in the study were marked in Figure 3 for ease of visualization, although a geographical differentiation was not set as part of the priors of the analysis. The frequency of each of the three clusters varied considerably among countries (Table 5). VNTR types whose genome belonged to a single cluster were deemed “pure” and typologies with admixed ancestry were identified in all clusters. At a general level, there were more VNTR types with admixed ancestry (43.3%) than pure types (Table 5). The largest proportion of VNTR types belonging to cluster I were reported in Brazil (41.9%), and in Spain for clusters II and III (34.8 and 20.3%, respectively). Mexico showed the largest proportion of admixed ancestry types (80.8%).

**Table 5:** Proportion and percentage of specimens belonging to clusters I-III and admixed ancestry of *M. hyopneumoniae* populations by country, with a 90% membership rate.

Country	Cluster I	Cluster II	Cluster III	Admixed ancestry
Argentina	24/65	6/65	10/65	25/65
	(36.9%)	(9.2%)	(15.4%)	(38.5%)
Belgium	8/31	4/31	3/31	16/31
	(25.8%)	(12.9%)	(9.7%)	(51.6%)
Brazil	83/198	24/198	27/198	64/198
	(42%)	(12.1%)	(13.6%)	(32.3%)
Mexico	3/26	1/26	1/26	21/26
	(11.6%)	(3.8%)	(3.8%)	(80.8%)
Spain	5/69	24/69	14/69	26/69
	(7.2%)	(34.8%)	(20.3%)	(37.7%)
United States	47/408	99/408	69/408	193/408
	(11.5%)	(24.3%)	(16.9%)	(47.3%)
Total	170/797	158/797	124/797	345/797
	(21.3%)	(19.8%)	(15.6%)	(43.3%)

## 4 Discussion

Bacterial population structures range from panmictic or non-clonal, to strictly clonal, but intermediate structures have also been recognized, such as epidemic (32). The different population genetic structures may be due to events like genetic recombination, geographical or ecological isolation, genetic drift, horizontal gene transfer, among others (32–35). While *Salmonella enterica* is a model of a clonal population, *Neisseria gonorrhoeae* and *Helicobacter pylori* are representative of non-clonal models (36). In the former, the same or very similar types of individuals can cause disease and/or outbreaks, and in the latter genetically unrelated individuals of a given type lead to malaise.

In the present study, the fact that more than 40% of the analyzed VNTR types belonged to admixed ancestry, three defined genetic clusters were identified, differences in diversity among countries was minimal, and no *p97*R1-*p146*R3 MLVA type was reported in all countries suggests that the genetic structure of *M. hyopneumoniae* is predominantly, but not exclusively, panmictic or non-clonal. On the other hand, the abundance of VNTR types, in addition to unique alleles identified in different countries strongly suggests that *M. hyopneumoniae* is a highly diverse microorganism.

Our findings indicate that three *M. hyopneumoniae* MLVA genetic clusters, which possibly evolved separately, are now seen combined globally, or at least in the analyzed countries. A similar pattern has been described for *M. bovis*, where three genetic clusters have been reported, with a preponderance of two (37). In the above mentioned study, the authors used MLST typing for 137 *M. bovis* isolates from 12 countries and observed the first cluster including isolates from multiple geographical origins (United Kingdom, Lithuania, Hungary, Germany, Italy, Israel, and Australia) and European origin only (Spain, Germany, France, and Lithuania). The second cluster including also isolates from several origins and all the strains from China and most of those of Israeli and Australian origin, and the third, including only two Israeli isolates (37). Moreover, similar patterns of geographical distribution of *M. bovis* isolates has been recognized using the MLVA typing method (38).

Numerous sequence-based typing approaches for *M. hyopneumoniae* have been developed, being MLST and MLVA the most commonly used currently. However, the use of one or the other techniques should not be arbitrary. While the MLST scheme, which like Multiple Locus Enzyme Electrophoresis (MLEE) detects DNA changes in conserved genome areas, MLVA was developed for high discrimination between closely related strains (39). This information, combined with the intermediate genetic structure of the agent might explain some incongruities reported to date. For instance, while some studies using MLST associated enzootic pneumonia (EP) with identical or very similar *M. hyopneumoniae* genotypes (12, 13, 40), others using MLVA typing reported a higher prevalence and severity of EP-like lung lesions with higher diversity of the pathogen (41). Particularly for *M. hyopneumoniae*, the use of the MLVA assay has been recommended for differentiation of strains with identical MLST sequence types (10). Considering that the MLST scheme is useful for phylogenetic analysis, but often not discriminative enough for outbreak detection (39), and that MLVA outcomes can be highly polymorphic, the use of both typing approaches in combination for EP outbreak studies could yield insightful information.

In spite of the fact that VNTR types with mixed ancestry were identified in the six analyzed countries, in Mexico, it reached almost 81%. The source of this type of VNTR could be due, for instance, to genetic drive or horizontal gene transfer. Future studies are needed for a better understanding of this phenomenon. In addition to this, Mexico is the only country among those analyzed in which a live attenuated *M. hyopneumoniae* vaccine has recently been licensed (42). Further longitudinal studies to evaluate the stability of VNTR types from *M. hyopneumoniae* hosted in pigs vaccinated with live vaccines should be conducted. Interestingly, the VNTR type of the J strain (9–18), the most used strain for inactivated vaccines against EP worldwide (18), did not match exactly any of the analyzed VNTR types. Similar types, namely 9–17, 9–19 and 8–17, were also identified in Argentina, Belgium, Spain and the United States. Contrary to what was observed in Mexico, in Brazil more VNTR types belonging to cluster I than with admixed ancestry were identified, suggesting that gene flow may be restricted between the Brazilian *M. hyopneumoniae* populations and those of other countries. In addition, if the Brazilian populations originated from a single source of infection and if not many types with a different genetic composition have entered the country, it could be expected for the *M. hyopneumoniae* populations to have a predominance of a single cluster with very little presence of the other genetic combinations, which has been observed in this study.

Results from this investigation suggest that most variation in *M. hyopneumoniae* types (61–90%) was observed within populations (countries). Although it has been suggested that a certain *M. hyopneumoniae* MLVA type predominates and persists within each herd (17, 19), circulating *M. hyopneumoniae* genotypes are abundant, at herd and even at pig level (15, 43, 44). The molecular variation among populations (7–10%) was probably due to the existence of unique alleles in some countries and to that a small number of *p97*R1 and *p146*R3 alleles were present in all countries. Results also indicated that for both loci in combination, and for the *p146*R3 locus alone, approximately 30% of variation was observed between regions (America vs. Europe), which was probably due to the high polymorphism of the *p146*R3 locus (6). A practical implication of this finding would be to use *p146*R3 together with other polymorphic loci when making comparisons between the two continents.

In conclusion, this study reinforces the concept that *M. hyopneumoniae* is a genetically diverse pathogen with limited clonality. When typed by MLVA, three well-defined populations or clusters and their combinations were identified. The major cause of genetic variation of *M. hyopneumoniae* might be within populations, recognizing the bias in the analyzed data, as not all countries were analyzed using the same number of farms, nor randomly. This study constitutes a starting point for future investigations to provide greater detail of the molecular epidemiological behavior of this pathogen. The difficulty in culturing and isolating *M. hyopneumoniae* strains and the scarcity of genomes available, continues to be a limitation for the application of whole genome sequencing (WGS). Analysis of WGS of this pathogen could offer greater insight into genetic variability than that of the comparison of a small number of loci.

## Data Availability

The original contributions presented in the study are included in the article/Supplementary material, further inquiries can be directed to the corresponding author/s.

## Footnotes

^1^ http://taylor0.biology.ucla.edu/struct_harvest/
