# Supplementary material for: Genetic structure and diversity of Mycoplasma hyopneumoniae based on a MLVA typing scheme
Source: Front Vet Sci. 2025 Jan 15;11:1510825. doi: 10.3389/fvets.2024.1510825 (PMC11776303; doi:10.3389/fvets.2024.1510825)
Supplement: Supplementary file 2 [file Data_Sheet_2.DOCX]

**Supplementary Table S2**. Allele frequencies by country (populations) for the p97R1 and p146R3 loci.

| **Locus** | **Allele**  **(TR)** | **Argentina** | **Brazil** | **Mexico** | **USA** | **Belgium** | **Spain** |
| --- | --- | --- | --- | --- | --- | --- | --- |
| ***p97*R1** | **1** | 0,000 | 0,000 | 0,000 | 0,000 | 0,065 | 0,000 |
|  | **2** | 0,000 | 0,005 | 0,000 | 0,002 | 0,000 | 0,000 |
|  | **3** | 0,000 | 0,167 | 0,000 | 0,027 | 0,000 | 0,000 |
|  | **4** | 0,000 | 0,000 | 0,000 | 0,020 | 0,000 | 0,000 |
|  | **5** | 0,000 | 0,000 | 0,077 | 0,005 | 0,000 | 0,000 |
|  | **6** | 0,138 | 0,005 | 0,000 | 0,022 | 0,000 | 0,000 |
|  | **7** | 0,000 | 0,076 | 0,000 | 0,047 | 0,129 | 0,000 |
|  | **8** | 0,369 | 0,020 | 0,000 | 0,039 | 0,065 | 0,174 |
|  | **9** | 0,015 | 0,152 | 0,231 | 0,248 | 0,161 | 0,362 |
|  | **10** | 0,062 | 0,071 | 0,577 | 0,034 | 0,129 | 0,014 |
|  | **11** | 0,323 | 0,152 | 0,038 | 0,135 | 0,065 | 0,290 |
|  | **12** | 0,000 | 0,338 | 0,000 | 0,076 | 0,258 | 0,072 |
|  | **13** | 0,092 | 0,010 | 0,038 | 0,100 | 0,000 | 0,014 |
|  | **14** | 0,000 | 0,005 | 0,000 | 0,042 | 0,065 | 0,014 |
|  | **15** | 0,000 | 0,000 | 0,000 | 0,074 | 0,065 | 0,043 |
|  | **16** | 0,000 | 0,000 | 0,000 | 0,098 | 0,000 | 0,000 |
|  | **17** | 0,000 | 0,000 | 0,000 | 0,025 | 0,000 | 0,014 |
|  | **18** | 0,000 | 0,000 | 0,000 | 0,005 | 0,000 | 0,000 |
|  | **19** | 0,000 | 0,000 | 0,000 | 0,002 | 0,000 | 0,000 |
|  | **20** | 0,000 | 0,000 | 0,038 | 0,000 | 0,000 | 0,000 |
| ***p146*R3** | **10** | 0,000 | 0,000 | 0,000 | 0,010 | 0,000 | 0,000 |
|  | **11** | 0,000 | 0,000 | 0,000 | 0,025 | 0,000 | 0,000 |
|  | **12** | 0,000 | 0,000 | 0,000 | 0,017 | 0,000 | 0,014 |
|  | **13** | 0,000 | 0,005 | 0,000 | 0,015 | 0,032 | 0,014 |
|  | **14** | 0,031 | 0,313 | 0,000 | 0,012 | 0,000 | 0,014 |
|  | **15** | 0,092 | 0,040 | 0,000 | 0,262 | 0,032 | 0,000 |
|  | **16** | 0,338 | 0,010 | 0,000 | 0,027 | 0,000 | 0,000 |
|  | **17** | 0,077 | 0,066 | 0,115 | 0,093 | 0,226 | 0,043 |
|  | **18** | 0,000 | 0,030 | 0,346 | 0,051 | 0,097 | 0,000 |
|  | **19** | 0,000 | 0,000 | 0,154 | 0,022 | 0,000 | 0,000 |
|  | **20** | 0,000 | 0,000 | 0,038 | 0,051 | 0,000 | 0,348 |
|  | **21** | 0,000 | 0,141 | 0,192 | 0,152 | 0,000 | 0,043 |
|  | **22** | 0,000 | 0,005 | 0,077 | 0,049 | 0,032 | 0,029 |
|  | **23** | 0,000 | 0,010 | 0,038 | 0,015 | 0,032 | 0,014 |
|  | **24** | 0,000 | 0,086 | 0,000 | 0,071 | 0,065 | 0,014 |
|  | **25** | 0,185 | 0,005 | 0,000 | 0,054 | 0,032 | 0,000 |
|  | **26** | 0,200 | 0,005 | 0,038 | 0,047 | 0,000 | 0,014 |
|  | **27** | 0,000 | 0,010 | 0,000 | 0,025 | 0,065 | 0,000 |
|  | **28** | 0,000 | 0,000 | 0,000 | 0,002 | 0,032 | 0,000 |
|  | **29** | 0,077 | 0,005 | 0,000 | 0,000 | 0,000 | 0,000 |
|  | **30** | 0,000 | 0,000 | 0,000 | 0,000 | 0,000 | 0,029 |
|  | **31** | 0,000 | 0,005 | 0,000 | 0,000 | 0,000 | 0,014 |
|  | **32** | 0,000 | 0,025 | 0,000 | 0,000 | 0,000 | 0,058 |
|  | **33** | 0,000 | 0,030 | 0,000 | 0,000 | 0,000 | 0,000 |
|  | **34** | 0,000 | 0,020 | 0,000 | 0,000 | 0,129 | 0,000 |
|  | **35** | 0,000 | 0,025 | 0,000 | 0,000 | 0,000 | 0,029 |
|  | **36** | 0,000 | 0,000 | 0,000 | 0,000 | 0,000 | 0,014 |
|  | **38** | 0,000 | 0,020 | 0,000 | 0,000 | 0,000 | 0,014 |
|  | **39** | 0,000 | 0,000 | 0,000 | 0,000 | 0,065 | 0,014 |
|  | **40** | 0,000 | 0,005 | 0,000 | 0,000 | 0,000 | 0,029 |
|  | **41** | 0,000 | 0,005 | 0,000 | 0,000 | 0,065 | 0,014 |
|  | **42** | 0,000 | 0,035 | 0,000 | 0,000 | 0,000 | 0,014 |
|  | **43** | 0,000 | 0,015 | 0,000 | 0,000 | 0,065 | 0,014 |
|  | **44** | 0,000 | 0,061 | 0,000 | 0,000 | 0,032 | 0,000 |
|  | **45** | 0,000 | 0,015 | 0,000 | 0,000 | 0,000 | 0,029 |
|  | **46** | 0,000 | 0,000 | 0,000 | 0,000 | 0,000 | 0,145 |
|  | **47** | 0,000 | 0,000 | 0,000 | 0,000 | 0,000 | 0,029 |
|  | **48** | 0,000 | 0,005 | 0,000 | 0,000 | 0,000 | 0,000 |

Shading indicates *p97*R1 and *p146*R3 alleles found in all countries.
